# The impact of uncertainties associated with MammoSite brachytherapy on the dose distribution in the breast

**DOI:** 10.1120/jacmp.v12i4.3464

**Published:** 2011-11-15

**Authors:** Saleh Bensaleh, Eva Bezak

**Affiliations:** ^1^ School of Chemistry & Physics University of Adelaide South Australia 5005; ^2^ Department of Medical Physics Royal Adelaide Hospital North Terrace South Australia 5000 Australia

**Keywords:** MammoSite, breast, contrast medium, Monte Carlo, TLD

## Abstract

The MammoSite radiation therapy system is a novel technique for treatment of patients with early‐stage breast cancer. It was developed to overcome the longer schedules associated with external‐beam radiation therapy. It consists of a small balloon (4 cm in diameter) connected to an inflation channel and a catheter for the passage of a high dose rate ${\;}^{192}\text{Ir}$ brachytherapy source. The device is placed into the tumor resection cavity and inflated with a mixture of saline and radiographic contrast agent to a size that fills the cavity. A high dose rate ${\;}^{192}\text{Ir}$ source is driven into the balloon center using a remote afterloader to deliver the prescribed dose at a point 1 cm away from the balloon surface. There are several uncertainties that affect the dose distribution in the MammoSite brachytherapy. They include source position deviation, balloon deformation, and the concentration of the contrast medium inside the balloon. The purpose of this study is to investigate the extent of the dose perturbation for various concentrations of the contrast medium in a MammoSite balloon using Monte Carlo simulations and thermoluminescent dosimetry. This study also combines the impact of these uncertainties on the MammoSite treatment efficacy. The current study demonstrates that the combined uncertainties associated with the MammoSite brachytherapy technique — up to the value of 2 mm balloon deformation, 1 mm source deviation, and 15% contrast concentration — have no impact on the tumor control probability.

PACS number: 87.53.‐j

## I. INTRODUCTION

In the management of early‐stage (I or II) breast cancer, breast‐conserving surgery followed by radiotherapy is an alternative treatment to mastectomy for patients who desire breast preservation. The radiation is presumed to eliminate any residual cancer cells near the surgical cavity. Different radiotherapy modalities are available, and their applications depend on the tumor type, location, and staging.

The MammoSite radiation therapy system (Hologic, Inc., Marlborough, MA), is a relatively new brachytherapy procedure for accelerated partial breast irradiation. It is being used as an alternative to whole breast external beam irradiation for treatment of patients with early‐stage breast cancer. There are recommendations regarding the patient selection for the MammoSite treatment. Generally, it is used for treatment of patients diagnosed with ductal carcinoma in situ (DCIS), invasive ductal carcinoma (IDC), and a primary tumor of size ≤3 cm. The main advantage of this technique is the high conformal dose deposition in the target volume and sparing of the organs at risk due to the influence of the inverse square law effect on the dose distribution around the source.

The MammoSite device consists of a small balloon (ranging from 4–6 cm in diameter) connected to a catheter with an inflation channel and another for the passage of a high dose rate (HDR) ${\;}^{192}\text{Ir}$ brachytherapy source. The device is placed inside the breast surgical cavity and inflated with a combination of saline and radiographic contrast medium. The ${\;}^{192}\text{Ir}$ source is positioned at the balloon center for the treatment duration to deliver the prescribed dose. If used as a monotherapy, the treatment with the MammoSite device is generally 34 Gy delivered in 10 fractions (3.4 Gy/fraction twice daily) at 1.0 cm from the balloon surface with a minimum of 6 hours between fractions on the same day.^(^ ^1^ ^,^ ^2^ ^)^

There are several sources of uncertainties that impact the MammoSite dose distribution in the breast. They include source position deviation, balloon deformation, and the concentration of the contrast medium inside the balloon. These parameters need to be investigated as they may have a significant impact on the treatment efficacy. Uncertainties in source position and balloon deformation will perturb the prescribed dose to the target volume. The impact of these two types of uncertainties on the MammoSite treatment effectiveness was previously investigated.^(^ ^3^ ^)^

In order to enhance image quality and balloon visibility for treatment planning procedures, contrast medium is used inside the balloon.^(^ ^1^ ^,^ ^2^ ^,^ ^4^ ^)^ When the balloon is filled with saline only, it is difficult to visualize and to make a treatment plan. It is therefore necessary to add contrast inside the balloon. Because the contrast materials typically contain elements with high atomic number (for example, iodine with Z=53), the balloon content can no longer be considered tissue or water equivalent. Most of the currently available treatment planning systems for brachytherapy estimate the dose in a patient using the precalculated dose matrices derived from the ${\;}^{192}\text{Ir}$ source positioned in a water phantom,^(^ ^5^ ^)^ and do not take variations in attenuation caused by the contrast medium into account.

Several studies have investigated the dose perturbation extent from the contrast medium within the balloon using Monte Carlo simulations and measurements.^(^ ^6^ ^–^ ^9^ ^)^ The experimental measurements were performed by placing the MammoSite balloon in a water tank and dose measurements were carried out with the use of an ion chamber.^(^ ^8^ ^)^ The size of the chamber would lead to averaging of the dose gradient, and the water tank would not represent patient geometry. The purpose of this study is to investigate the extent of the dose perturbation for various concentrations of the contrast medium in a MammoSite balloon using Monte Carlo (MC) simulations and thermoluminescent dosimetry. This study also estimates the impact of the combined uncertainties associated with the MammoSite brachytherapy on the tumor control probability.

## II. MATERIALS AND METHODS

### A. Uncertainties in contrast concentration

#### A.1 Dose measurements using TLDs

TLD‐100 chips were used to measure the dose falloff at various distances from the balloon surface resulting from the presence of various contrast concentrations within the MammoSite balloon. The measurements were carried out in a tissue‐equivalent breast phantom. Before using the TLDs for dose measurements, their individual sensitivity correction factor was determined by irradiating them to a uniform dose using a linear accelerator. The process of TLD measurement is discussed in more detail in the following sections.

##### A.1.1 LiF TLD

The current investigation of the extent of the dose perturbation for various concentrations of the contrast medium in a MammoSite balloon is performed using LiF:Mg, Ti TLD‐100 chips (Krakow, Poland). These TLDs are composed of lithium fluoride (LiF) doped with magnesium (Mg) and titanium (Ti). They have several useful features. Their effective atomic number $\left(Z_{\text{eff},\text{LiF}}\right)$ is equal to 8.2, which is close to the effective atomic number of soft tissue $\left(Z_{\text{eff},\text{tissue}}\right)$ of 7.4, and makes this type of TLD suitable for clinical radiotherapy. They are insensitive to light and the thermal fading is small (<5% in 1 year). They are dose rate independent. The dimensions of the TLD‐100 chips are approximately 3.2 mm× 3.2 mm×0.38 mm. The small thickness of the TLDs allows for high dose resolution measurements in the direction of rapid dose gradient typical for brachytherapy dose distributions.

##### A.1.2 TLD annealing cycle

In the current study, 50 TLD chips were annealed in the TLD annealing oven (Victoreen Instruments, Elimpex‐Medizintechnik, Moedling, Austria) before irradiating. The annealing was performed to erase any previous irradiation effect and to achieve reproducible results.^(^ ^10^ ^)^ In this work, the TLDs were annealed using the following procedure: 400°C for 1 hour and then cooled between aluminum blocks for 2 hours, then annealed for 20 hours at 80°C and cooled between aluminum blocks.

##### A.1.3 Sensitivity correction factor (SCF)

In order to account for individual response differences between TLDs, their individual sensitivity correction factors were measured. The TLDs were exposed lying on the surface of a $25.5\times 25.5\times 1\;{\text{cm}}^{3}$ solid water phantom with holes cut to fit a single TLD chip. A buildup layer of solid water of 1.3 cm thickness was used to place the chips at the depth of maximum dose. All TLDs were irradiated to a uniform dose of 1 Gy using a 6 MV photon beam from a Varian iX linear accelerator (Varian Medical Systems, Palo Alto, CA). This dose was given at a depth of maximum dose (1.3 cm) in a $10\times 10\;{\text{cm}}^{2}$ field size at a source‐to‐surface distance (SSD) of 100 cm.

The TLDs were read after a 24 hour waiting period. This waiting time assured that any residual thermoluminescence from the short half‐life peaks would have no significant contribution to the signal.^(^ ^11^ ^,^ ^12^ ^)^ The TLD signal was read out in a Harshaw Model 3500 Automatic TL reader (Thermo Fisher Scientific, Waltham, MA). A temperature between 50°C to 300°C at a constant heating rate of $10{}^{\circ}C\;s^{-1}$ was applied. During every reading cycle, several unirradiated chips were used for background subtraction. After readout, all the TLD chips were again subjected to the above annealing procedure. The whole set of the chips was subjected to three cycles of annealing, irradiation, and readout. The TLD readings were transferred to a spreadsheet for subsequent analysis.

The SCF compensates for the variation in sensitivity among the TLDs. The SCF was measured three times to determine the stability and reproducibility of the chip responses. The mean value of these measurements was used to obtain the SCF. The individual SCF of TLD chips was calculated using the following equation:

(1) $$SCF_{i}=\frac{\bar{R}}{R_{i}}$$

where

(2) $$\bar{R}=\frac{1}{N}\sum_{i=1}^{N}R_{i}-R_{BKG}$$

where ${\mathrm{SCF}}_{i}$ is the sensitivity correction factor for TLD chip $\#i(i=1,2,\ldots ),\bar{R}$ is the average integral readings of the whole batch of TLDs for a specific irradiation cycle, *N* is the total number of the irradiated chips, $R_{i}$ is the gross thermoluminescence reading, and $R_{\mathrm{BKG}}$ is the background readings of the unexposed chips.

##### A.1.4 Dose linearity range for TLD chips

The dose response linearity of TLD‐100 chips was investigated by exposing the detectors to different doses from 0.01 to 10 Gy. For each dose, three TLDs were irradiated in the solid water phantom described previously. The TLDs were irradiated with a 6 MV beam from Varian iX linear accelerator at depth of dose max for a $10\times 10\;{\text{cm}}^{2}$ field at 100 cm SSD.

After irradiation, the TLD chips were read the following day. Each reading was corrected for sensitivity and for background. The thermoluminescent readout of each detector was calculated, with correction for $R_{\mathrm{BKG}}$, *SCF, BCF* and as follows:

(3) $$TL_{i}=(R_{i}-R_{BKG})\cdot SCF_{i}\cdot BCF$$

where *BCF* is the batch correction factor which accounts for the change of the readout of each TLD chip due to changes in the heating characteristics (irradiation, reading, and annealing). BCF is obtained using the following relation:

(4) $$BCF=\frac{{\bar{R}}_{\exp ected}}{{\bar{R}}_{measured}}$$

From the previous section, the mean reading of 5 TLD chips, randomly selected, was reported. The SCF for these chips was calculated using Eqs. (1) and (2) above. The mean reading of these detectors is the expected readout $\left({\bar{R}}_{\exp ected}\right)$. During the measurement of the dose linearity response of the TLD chips, the randomly selected TLD chips were irradiated again to the same uniform dose conditions as in the previous section. The thermoluminescent reading of these chips was corrected for sensitivity (*SCF*) and for background $\left(R_{\mathrm{BKG}}\right)$. The mean reading of these dosimeters is called $\left({\bar{R}}_{measured}\right)$. The ratio of mean readings for these TLDs is called the batch correction factor (BCF), as given in Eq. (4).

#### A.2 Phantom design

In the current study, a breast phantom was constructed in‐house from near tissue equivalent materials (50% paraffin, 50% bees wax, and with relative density of 0.926) and was used for investigation of the perturbing effects of different contrast concentration in the MammoSite balloon (4 cm diameter) on the dose distribution in the breast. The phantom should simulate full‐scatter geometry. The phantom was split into two halves along the central axis to allow for placement of the MammoSite balloon and TLDs chips. Holes were drilled in the breast phantom to hold the TLDs at different distances from the balloon surface. The TLDs provided experimental measurement of the dose at various depths from the balloon surface along the radial axis. The breast‐equivalent phantom was placed into position on a RANDO anthropomorphic phantom (The Phantom Laboratory, Salem, NY).

#### A.3 Treatment simulation

**Figure 1:**
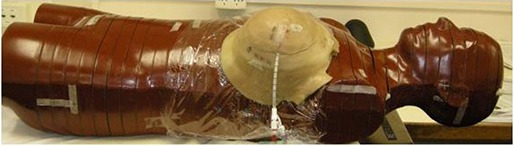
The inflated balloon placed inside the breast tissue‐equivalent phantom and attached to the Rando anthropomorphic phantom.

The MammoSite balloon was first filled with saline only to the desired volume and the two halves of the phantom were joined together as shown in Fig. 1. Computed tomography images of the breast phantom were then obtained from a CT scanner (Philips Medical Systems Ltd, Stevenage, UK) using contiguous slices of 3 mm thickness. The saline in the balloon was then replaced with an equal volume of a 100% contrast solution and a CT scan was acquired. The contrast medium used in this investigation is Ultravist 370 (Bayer Health Care Pharmaceutical Inc., Germany) which is a water‐soluble radiopaque medium. It has a molecular composition of $C_{18}H_{24}I_{3}N_{3}O_{8}$ and a molecular weight of 790.91, of which 48.13% iodine content by weight and density of $1.409\;g\;{\text{cm}}^{-3}$. The contrast solutions were carefully prepared and kept in a dark environment to avoid recrystallization. The filling and scanning process was repeated for a balloon filled with saline and 15% radiographic contrast concentration, and for saline and 50% radiographic contrast concentration. For HDR MammoSite brachytherapy treatment planning, the CT images were transferred to the Plato brachytherapy planning system (Plato BPS v14.3.2, Nucletron B‐V., Veenendaal, The Netherlands).

#### A.4 MammoSite treatment

**Figure 2:**
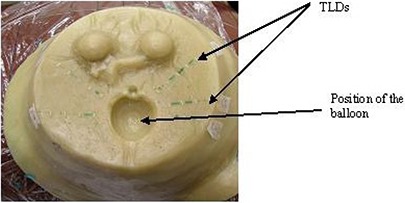
Positions of TLDs at various distances from the balloon surface.

To measure the effect on dose distribution from the contrast medium, the balloon was first filled with saline only and the TLD chips were placed in the breast phantom at various distances from the balloon surface along the radial axis, as shown in Fig. 2. The detectors were positioned in a direction perpendicular to the catheter to avoid averaging of the dose along the gradient. The two halves of the phantom were then joined together. The MammoSite treatment plan, which contains the source position and the dwell time information, was sent to the treatment control unit, the Nucletron microSelectron HDR afterloder (Nucletron International, Veenendaal, The Netherlands), to be delivered. It is important that the HDR source is positioned accurately at the balloon center to deliver the prescribed dose. The MammoSite treatment consisted of prescribing 3.4 Gy at 1 cm from the surface of the balloon. A microSelectron HDR unit was used in the current study. The HDR source had an activity of approximately 10 Ci (370 GBq) during this study.

Following irradiation, The TLD chips were read according to the procedure explained previously. They were then annealed and reused again. The TLD measurement procedure was repeated for a balloon filled with contrast only, and saline and 15% contrast, saline and 50% contrast. For each measurement, several TLD chips were set aside without any radiation and used for background subtraction. The thermoluminescent readout of each detector was calculated using Eq. (3).

**Figure 3:**
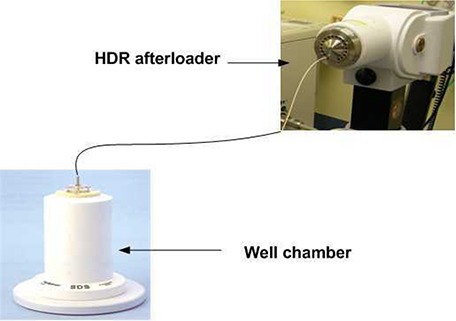
Experimental measurement setup for irradiating the TLDs to HDR source.

The BCF for HDR exposure was determined in a similar manner, as explained previously, by exposing the TLDs to the HDR source instead of 6 MV X‐rays. Five TLD chips were positioned at the center of the well chamber and the HDR source was driven at the center of the well chamber to deliver a dose of 5 Gy. Figure 3 is a representation of the experimental setup for exposing the TLDs to HDR source. In Fig. 3, the TLDs are not seen because they were placed within an insert for HDR source. Equation (4) was used to calculate the BCF.

#### A.5 Dose reduction factor

The readouts of the TLDs were used to measure the dose reduction factor (DRF) resulting from the presence of contrast solution in the MammoSite balloon. The DRF is defined as the dose with the contrast in the balloon to the dose with saline only in the balloon. It was calculated using the following relation:

(5) $$DRF=\frac{D(E,d,x,C)}{D(E,d,x,0)}$$

where *D* is the dose expressed as a function of photon energy (E), the diameter of the balloon (d), the distance from the balloon surface (x), and concentration of the contrast concentration (C).

#### A.6 Monte Carlo simulation

The EGSnrc Monte Carlo system^(^ ^13^ ^)^ is used in the current study. The EGSnrc is a new version of the EGS4 (Electron‐Gamma‐Shower) system that is used for the simulation of the transport of electrons and photons through a media over an energy range of a few keV up to several TeV.^(^ ^14^ ^)^ It follows the interaction of the transported particles until their energies fall below the given threshold. The dose calculation in the current work was performed with the DOSXYZnrc^(^ ^15^ ^)^ Monte Carlo code. This code was chosen in the current work because the dose calculations were performed using CT images of the MammoSite phantom. Monte Carlo simulations, done for investigating the dose perturbation for various contrast concentrations in the MammoSite balloon, are addressed in the following sections.

The CT images of the MammoSite phantom were also imported to the EGSnrc Monte Carlo code. The CT images were converted to a specific file format (*.egsphant) to be used for Monte Carlo dose calculations. The CTCREATE code,^(^ ^16^ ^)^ available with the EGSnrc Monte Carlo package, was used for the CT dataset conversion.

The radiographic contrast was modeled in EGSnrc by specifying the percentage weight of each component and the physical density of the contrast, as shown in Table 1. These compositions and densities were put into the EGSnrc code and used for dose calculations. The material data were created using the egs‐gui^(^ ^17^ ^)^ code available with the EGSnrc Monte Carlo package.

**Table 1:** Compositions and densities of the simulated contrast solutions.

*% Contrast*	*% Carbon*	*% Hydrogen*	*% Iodine*	*% Nitrogen*	*% Oxygen*	*Density (g/cc)*
15	4.16	9.97	7.22	0.80	77.92	1.06
50	13.66	7.12	24.06	2.66	52.49	1.20
100	27.31	3.06	48.13	5.31	16.18	1.41

The CT dataset for a balloon (4 cm diameter) filled with saline only, contrast only, and saline and different contrast concentrations, was used for dose calculations using the DOSXYZnrc Monte Carlo user code. In the DOSXYZnrc code, the ${\;}^{192}\text{Ir}$ brachytherapy source was approximated as a uniformly isotropically radiating parallelepiped source.^(^ ^3^ ^)^ The γ‐ray spectrum for the ${\;}^{192}\text{Ir}$ was taken from a published report.^(^ ^18^ ^)^ The spectrum consists of 34 energy bins ranging from 0.060 to 0.885 MeV. In the Monte Carlo simulation, electrons and photons were tracked until their energies reach the predefined cutoff values ECUT and PCUT, respectively. These Monte Carlo parameters were set to AE=ECUT=0.521 MeV, AP=PCUT=0.06 MeV.^(^ ^6^ ^)^

In each simulation, the modeled ${\;}^{192}\text{Ir}$ source was placed at the center of the MammoSite balloon (4 cm in diameter) with its axis aligned with the balloon axis. The voxel size of the three‐dimensional scoring geometry was 0.15 cm ×0.15 cm ×0.15 cm. For each simulation, 4 × 107 particles were tracked resulting in a statistical uncertainty of 1.8% at 1 cm from the balloon surface. This resulted in a CPU running time between 80–119 hours for the different Monte Carlo runs. The STATDOSE Monte Carlo user code,^(^ ^19^ ^)^ available with the EGSnrc Monte Carlo system, was used to extract the dose distributions in the CT dataset at various distances from the balloon surface. To determine the extent of dose perturbation for different contrast concentrations in the MammoSite balloon (4 cm diameter), the dose was normalized to that with a balloon filled with saline only. Monte Carlo simulations were also performed for balloons with 5 cm and 6 cm diameters.

### B. Uncertainties in source position and balloon deformation

The impact of the source position on the dose distribution of the MammoSite was investigated using Monte Carlo simulation. The Monte Carlo dose simulation was based on CT datasets. The ${\;}^{192}\text{Ir}$ source was first placed at the center of the MammoSite balloon and up to $2\times 10^{8}$ incident particles were tracked resulting in an overall relative statistical uncertainty of less than 2% at the prescription point. The ${\;}^{192}\text{Ir}$ source was then shifted up to 5 mm by a margin of 1 mm and the same numbers of particles were simulated. The STATDOSE Monte Carlo user code was used to extract dose profiles at 1 cm from the balloon surface resulting from source shift simulations. These extractions were used to investigate the impact of source position on the prescribed dose.

The CT images were also sent to the Plato brachytherapy planning system for the balloon position and shape verification, treatment planning, and dose calculations. The balloon‐cavity conformance, balloon symmetry, and the distance from the surface of the balloon to the skin were measured and examined on the CT images. The planning target volume (PTV) was defined as the volume of tissue extending 10 mm (prescription point) from the balloon surface. The balloon was not considered to be part of the PTV. A treatment plan was developed with the ${\;}^{192}\text{Ir}$ brachytherapy source at the balloon center, and a dose of 3.4 Gy was prescribed to a 10 mm radial distance from the surface of the balloon. Dose volume histograms (DVHs) were generated for the PTV with the ${\;}^{192}\text{Ir}$ source positioned at the balloon center. The balloon was deformed by up to 4 mm in 1 mm steps and similarly 3.4 Gy was delivered at the prescription point. DVHs were constructed for each balloon deformation treatment plan. The DVHs were imported from the treatment planning system and used for tumor control probability (TCP) calculations. The TCP was calculated from the cell surviving fraction using the Poisson hypothesis.^(^ ^20^ ^–^ ^23^ ^)^

DVHs were obtained from the Plato brachytherapy treatment planning system. The Plato planning system does not account for the attenuation caused by the contrast content inside the balloon; hence Monte Carlo simulations were performed to determine the dose reduction values caused by the contrast. The Monte Carlo simulations were performed for a range of balloon radii and various contrast concentrations. These dose reduction values were then considered as correction factors to the prescribed dose, and DVHs were recalculated accordingly to correct for the attenuations caused by the different contrast concentrations.

The DVHs were then used to calculate the TCP using the following Poisson hypothesis:^(^ ^23^ ^)^

(6) $$TCP=e^{-k\cdot S}$$

where *k* is the cell number of tumor clonogens and *S* is the surviving fraction.

The surviving fraction was calculated using the following relations:^(^ ^20^ ^,^ ^22^ ^,^ ^24^ ^)^

(7) $$S=e^{-\{\alpha D(1+Gd/\alpha /\beta )\}-\gamma T}$$

(8) $${\left(BE_{ff}D\right)}_{i}=D_{i}[1+Gd_{i}/\alpha /\beta ]$$

where α and β are radiobiological parameters, *d* is the dose per fraction, *G* is the dose protraction factor which accounts for both the dose rate and repair of sublethal damage, γ is the effective tumor‐cell repopulation rate, *T* is effective treatment, and $BE_{ff}D$ is the biological effective dose.

The surviving fraction was calculated for each bin of the $BE_{ff}D$‐based differential DVH using equations. For the MammoSite treatment, each fraction is delivered with approximately constant dose rate within short time, the protraction factor G in Eq. (7) was calculated by the following formula:^(^ ^24^ ^,^ ^25^ ^)^

(9) $$G=\frac{2}{n\mu T_{f}}\left[1-\frac{1}{\mu T_{f}}(1-e^{-\mu T_{f}})\right]$$

Here, *n* is the number of dose fractions, μ is the repair rate of tumor cells $\left(h^{-1}\right)$ and $T_{f}$ is the dose delivery time (h). The values of the parameters used to calculate S and TCP were obtained from the literature,^(^ ^26^ ^,^ ^27^ ^)^ as follows: $T_{f}=0.17h;\;k=200,\;\alpha =0.3\;Gy^{-1};\;\beta =0.03\;Gy^{-2};\;T_{d}=15$ days, $T_{\overset{1}{\;}/\underset{2}{\;}}=1\;h$, and $\mu =In(2)/T_{\overset{1}{\;}/\underset{2}{\;}}=0.69\;h^{-1}$. The TCP was calculated from DVHs that were constructed for each balloon deformation/source deviation treatment plan.

## III. RESULTS & DISCUSSION

### A. TLD calibration

The individual SCFs of each TLD‐100 chip were determined. It was found that the maximum SCF is 1.06, the minimum SCF is 0.96, and the mean value is 1.001 with standard deviation of 1.3%. The dose response curve for TLD‐100 chips was linear up to about 6 Gy; the mean relative deviation over the measurements was 1.9% with a range of 1.1%–2.6%.

### B. Phantom measurements using TLDs

**Figure 4:**
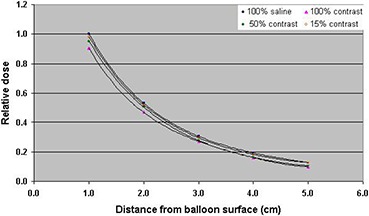
TLD measurements of variation of dose as a function of distance for a balloon filled with saline only, contrast only, saline and 50% contrast concentration, and saline and 15% contrast concentration. The measurement uncertainty with TLDs was within 3%.

Previous studies measured the dose reduction resulting from the use of contrast solution in the balloon, using a parallel plate ion chamber with larger diameter.^(^ ^6^ ^,^ ^8^ ^)^ These studies were done by placing the MammoSite balloon in a scanning water tank. The current study was performed by placing the MammoSite balloon inside a designed breast phantom made from tissue‐equivalent material, so it would resemble patient geometry. The TLD chips used in the current study had the advantages of small size (0.38 mm thickness) and dose rate independence which would make them good detectors for dose measurements with the HDR source. Due to the high‐dose gradient, the TLD chips were aligned in the breast phantom perpendicular to the source. This TLD orientation would minimize the dose averaging effect. The dose reduction resulting from the use of high atomic number contrast (in the MammoSite balloon) can cause considerable uncertainty (see Fig. 4). The relative dose was normalized to that with balloon filled with saline only. The measurement uncertainty with TLD chips was within 3%.

**Figure 5:**
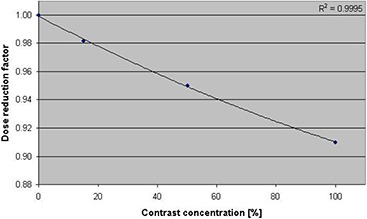
Dose reduction factor (at 1 cm from balloon surface) attributable to attenuation from various contrast concentrations in the MammoSite balloon.

To measure the extent of dose perturbation for various contrast concentration in the MammoSite balloon, the dose was normalized to that with balloon filled with saline only. The balloon filled with 100% contrast medium resulted in up to 10% dose perturbation at the prescription point due to attenuation of the radiation. The variation of dose with distance from the balloon surface, for the saline‐filled balloon with 50% contrast concentration, resulted in approximately 5% reduction in the dose at the prescription point, while a 15% contrast concentration produced nearly 2% dose reduction at the same point. The percentage difference in the dose reduction increased with increasing contrast concentration. The difference between the saline curve (no contrast medium) and the other curves (with contrast medium) indicates the dosimetric effect due to attenuation by the contrast medium. The dose reduction factor (DRF) value as a function of various contrast concentration levels is shown in Fig. 5. The DRF is larger for higher contrast concentrations.

### C. Monte carlo simulations

**Figure 6:**
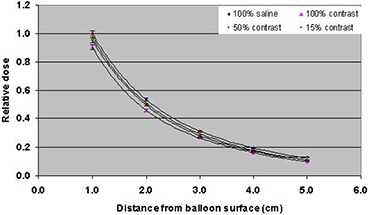
Monte Carlo simulations of variation of dose as a function of distance for a balloon filled with saline only, contrast only, saline and 50% contrast concentration, saline and 15% contrast concentration. The uncertainty in Monte Carlo calculation was within 1.8% at 1 cm from the balloon surface.

**Figure 7:**
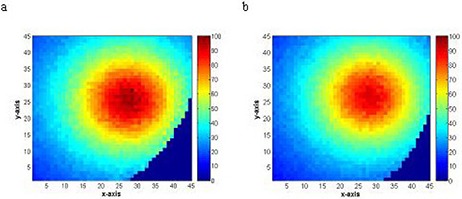
Monte Carlo dose distribution for balloon filled with (a) saline only, and (b) contrast only. The contrast inside the balloon resulted in 10% dose reduction at 1 cm (prescription point) from the balloon surface.

Monte Carlo simulations were used to calculate the radial axis dose distributions using CT data of a breast phantom at various distances of 1 cm to 5 cm from the surface of the balloon. The Monte Carlo results of dose perturbation at the prescription point are shown in Figs. 6 and 7 for a balloon diameter of 4 cm and contrast concentrations of 0%, 15%, 50%, and 100% by volume. The dose was normalized to that with balloon filled with saline only. One hundred percent (100%) contrast concentration reduced the dose at the prescription point by 10%; 15% contrast concentration produced about 2% dose reduction at the prescription point. The Monte Carlo simulation was validated with TLD measurements and the results of the two methods were in agreement.

Additional Monte Carlo simulations were performed for balloons of 5 cm and 6 cm diameters and contrast concentrations of 100%, 50%, and 15%. Table 2 shows the dose reduction values for range of balloon radii at 1 cm away from the balloon surface. The dose reduction at 1 cm for the balloon surface was 0.91 for the smallest balloon diameter of 4 cm filled with 100% contrast concentration. It was 0.886 for the largest balloon diameter of 6 cm at the same distance and 100% contrast concentration. For 15% contrast concentration, the dose reduction values were 0.982, 0.964, and 0.955 for balloon diameters of 4, 5, and 6 cm, respectively. The dose reduction factor is larger for larger balloon sizes and higher contrast concentrations. This is as expected, as photon attenuation will be affected more if they have to penetrate larger volumes of contrasts.

**Table 2:** The dose reduction factor values at the prescription point for various balloon diameters and contrast concentrations.

		*Balloon diameter (cm)*	
*Contrast Concentration (%)*	*4*	*5*	*6*
0	1	1	1
100	0.910	0.886	0.862
50	0.950	0.937	0.922
15	0.982	0.964	0.955

### D. Comparison between simulations and measurements

**Figure 8:**
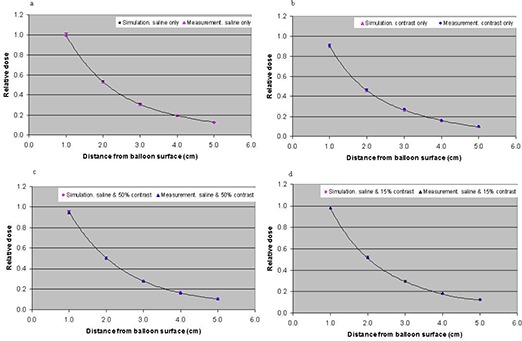
Comparison of Monte Carlo simulation and TLDs measurements showing the variation of dose as function of distance from the balloon surface for a balloon filled with (a) saline only, (b) 100% contrast concentration only, (c) saline with the addition of 50% contrast concentration, and (d) saline with the addition of 15% contrast concentration. The uncertainties in Monte Carlo calculation and TLD measurements were 1.8% and 3%, respectively.

A direct comparison of the TLD‐measured relative dose and the Monte Carlo‐computed dose as a function of distance from the balloon surface, for balloons filled with saline and different contrast concentrations, is shown in Fig. 8. The dose pattern shows a sharp falloff as a function of distance from the balloon surface. The measured and calculated dose is in agreement within the measurement uncertainty and Monte Carlo statistical errors.

### E. Combination of uncertainties

Uncertainty in source position had a pronounced impact on the MammoSite treatment dose. A 1 mm deviation in the source location results in portions of the treated volume at the prescription distance (i.e., 1 cm from the balloon surface) receiving less than the prescribed dose by 7% for a balloon diameter of 4 cm and by 5% for balloon diameters of 5 and 6 cm. It also results in other regions of the PTV receiving about 9% higher dose for a balloon diameter of 4 cm, and ~7% dose increase for balloon diameters of 5 and 6 cm. A 2 mm source deviation resulted in portions of the treated volume receiving about 14%, 11%, and 11% less than the prescribed dose (for balloon diameters of 4, 5, and 6 cm, respectively), whereas other regions of the treated volume received about 19%, 14%, and 14% higher dose (for balloon diameters of 4, 5 and 6 cm respectively) at the prescription point. This could expose the skin tissue to excessive dose especially in cases where the balloon–skin distance is at minimum. The data in Table 3 represent the effect of source shift on the dose at 1 cm from the balloon surface. The zero shift point represents the dose calculated for the ${\;}^{192}\text{Ir}$ source situated in the center of the balloon. The dose difference due to source shift is less for larger balloon sizes, compared to a 4 cm balloon diameter, due to exponential shape of dose gradient with shallower dose falloff at larger distances from the source center.

**Table 3:** Dose enhancement and reduction ratios as a result of source deviations for various balloon diameters.

	*Dose Enhancement Ratio Balloon diameter (cm)*			*Dose Reduction Ratio Balloon diameter (cm)*		
*Source Shift (mm)*	*4*	*5*	*6*	*4*	*5*	*6*
0	1.0	1.0	1.0	1.0	1.0	1.0
1	1.09	1.07	1.07	0.93	0.95	0.95
2	1.19	1.14	1.14	0.86	0.89	0.89
3	1.27	1.22	1.20	0.81	0.84	0.85
4	1.39	1.29	1.27	0.77	0.79	0.79
5	1.52	1.36	1.33	0.71	0.73	0.74

The American Association of Physicists in Medicine (AAPM) Task Group 40 recommendation for intracavitary brachytherapy allows for ±15% in the delivery of the prescribed dose contributing from all possible sources of uncertainties.^(^ ^28^ ^)^ The DVHs and Monte Carlo simulations were used to estimate the combined impact of uncertainties in MammoSite brachytherapy on the TCP. The TCP results for the combined MammoSite uncertainties are summarized in Table 4. The TCPs calculated for three different balloon sizes are similar (within the estimated uncertainty), as the effects of contrast concentration (the effect increases as a function of the balloon diameter) and balloon deformation (dose perturbation effect decreases as a function of the balloon diameter) partially counteract each other.

**Table 4:** TCP resulting from the combined uncertainties encountered in the MammoSite technique.

*Balloon Deformation (mm)*	*Contrast Concentration (%)*	TCP%(±2%) *Balloon Diameter (cm)*		
		*4*	*5*	*6*
0	15	99	99	99
0	50	98	98	98
1	15	96	97	97
1	50	95	95	96
2	15	94	93	94
2	50	90	89	90
4	15	74	74	74
4	50	70	69	70

The TCP, with the source at the balloon center, spherical balloon shape, and 15% contrast concentration inside the balloon, was about 99% for all balloon sizes. The TCP, with 1 mm balloon deformation and 15% contrast concentration inside the balloon, was about 96%. The TCP, with 2 mm balloon deformation and 15% contrast concentration inside the balloon, was reduced by 6%. The TCP, with 4 mm balloon deformation and 15% contrast concentration inside the balloon, was reduced by 26%. The TCP, with the source at the balloon center, spherical balloon shape, and 50% contrast concentration inside the balloon, was about 98%. The TCP, with 1 mm balloon deformation and 50% contrast concentration inside the balloon, was about 95%. The TCP, with 2 mm source deviation or balloon deformation and 50% contrast concentration inside the balloon, was reduced by 10%. The TCP, with 4 mm balloon deformation and 50% contrast concentration inside the balloon, was reduced by more than 30%.

## IV. CONCLUSIONS

This represents the first study performed on a humanoid phantom with small thickness TLD chips, as opposed to other studies carried out in a water tank with a large ion chamber. In addition, the Monte Carlo simulations were based on CT data which resembles patient geometry, as opposed to other studies where the Monte Carlo calculations were performed in a water phantom.

The dose reduction resulting from the use of high atomic number contrast (Iodine) can cause considerable uncertainty in the MammoSite dose. As revealed by Monte Carlo simulations and TLD measurements, the contrast media inside the MammoSite balloon resulted in a reduction in dose at the prescription point. The magnitude of dose reduction depends on the concentration of contrast. The dose perturbation is larger for higher contrast concentration. A 15% contrast concentration caused between 2% and 5% dose reduction at 1 cm from the balloon surface (depending on the balloon diameter), and 100% contrast concentration produced between 10% to 14% dose reduction at the same point. Based on the current investigation, we suggest that the amount of radiographic contrast used during MammoSite breast brachytherapy should be minimized (15% or less) to avoid potential significant reduction in the delivered dose.

In conclusion, the combined uncertainties associated with the MammoSite brachytherapy technique — to the value of a 2 mm balloon deformation, 1 mm source deviation, and 15% contrast concentration — have no impact on the tumor control probability for all manufactured balloon sizes.
